# Lethal Infection of Lassa Virus Isolated from a Human Clinical Sample in Outbred Guinea Pigs without Adaptation

**DOI:** 10.1128/mSphere.00428-19

**Published:** 2019-09-25

**Authors:** Junki Maruyama, John T. Manning, Elizabeth J. Mateer, Rachel Sattler, Natalya Bukreyeva, Cheng Huang, Slobodan Paessler

**Affiliations:** a Department of Pathology, the University of Texas Medical Branch, Galveston, Texas, USA; U.S. Centers for Disease Control and Prevention

**Keywords:** Lassa virus, guinea pig, animal model, clinical isolate

## Abstract

Lassa virus (LASV), a member of the family *Arenaviridae*, is the causative agent of Lassa fever. Lassa virus is endemic in West African countries, such as Nigeria, Guinea, Liberia, and Sierra Leone, and causes outbreaks annually. Lassa fever onset begins with “flu-like” symptoms and may develop into lethal hemorrhagic disease in severe cases. Although Lassa virus is one of the most alarming pathogens from a public health perspective, there are few licensed vaccines or therapeutics against Lassa fever. The fact that animal models are limited and the fact that mostly laboratory-derived viruses are used for studies limit the successful development of countermeasures. In this study, we demonstrated that the LASV isolate LF2384-NS-DIA-1 (LF2384), which was directly isolated from a serum sample from a fatal human Lassa fever case in the 2012 Sierra Leone outbreak, causes uniformly lethal infection in outbred Hartley guinea pigs without virus-host adaptation. This is the first report of a clinically isolated strain of LASV causing lethal infection in outbred guinea pigs. This novel guinea pig model of Lassa fever may contribute to Lassa fever research and the development of vaccines and therapeutics.

**IMPORTANCE** Lassa virus, the causative agent of Lassa fever, is a zoonotic pathogen causing annual outbreaks in West African countries. Human patients can develop lethal hemorrhagic fever in severe cases. Although Lassa virus is one of the most alarming pathogens from a public health perspective, there are few available countermeasures, such as antiviral drugs or vaccines. Moreover, the fact that animal models are not readily accessible and the fact that mostly laboratory viruses, which have been passaged many times after isolation, are used for studies further limits the successful development of countermeasures. In this study, we demonstrate that a human isolate of Lassa virus causes lethal infection uniformly in Hartley guinea pigs. This novel animal model of Lassa fever may contribute to Lassa fever research and the development of vaccines and therapeutics.

## Abstract (precis)

Lassa virus, the causative agent of Lassa fever, is a zoonotic pathogen causing annual outbreaks in West African countries. Human patients can develop lethal hemorrhagic fever in severe cases. Although Lassa virus is one of the most alarming pathogens from a public health perspective, there are few available countermeasures, such as antiviral drugs or vaccines. Moreover, the fact that animal models are not readily accessible and the fact that mostly laboratory viruses, which have been passaged many times after isolation, are used for studies further limits the successful development of countermeasures. In this study, we demonstrate that a human isolate of Lassa virus causes lethal infection uniformly in Hartley guinea pigs. This novel animal model of Lassa fever may contribute to Lassa fever research and the development of vaccines and therapeutics.

## OBSERVATION

Lassa virus (LASV), a member of the family *Arenaviridae*, is the causative agent of Lassa fever (LF). LASV is a zoonotic pathogen maintained in the rodent *Mastomys natalensis* as its natural reservoir (1). Humans are infected with LASV after exposure to the urine or feces of infected rodents or through direct contact with bodily secretions of a human LF patient. LF onset begins with “flu-like” symptoms. In severe cases, LF progresses into hemorrhagic disease with facial edema, high fever, and bleeding from mucosal membranes and gastrointestinal tracts. Death may occur within 2 weeks after the onset of initial symptoms due to multiorgan failure (2). Additionally, one-third of Lassa fever survivors develop sensorineural hearing loss, which is often permanent (3). LASV is endemic in West African countries, such as Nigeria, Guinea, Liberia, and Sierra Leone, and outbreaks occur annually. However, there are no licensed vaccines against LF. The antiviral drug Ribavirin is the only therapeutic option, although it must be administered early in the disease development (4). An estimated 37.7 million people are at risk of contracting LASV, emphasizing the critical need for the development of safe and effective vaccines and therapeutics (5). LASV must be handled at biosafety level 4 (BSL-4) due to its high pathogenicity. This is one of the largest barriers for the development of preventive or therapeutic approaches against LF. Furthermore, animal models of LF are limited, especially models that utilize clinically isolated viruses. The nonhuman primate (NHP) model, which is the gold standard for LF studies, correlates well with human LF (6, 7). However, there is concern about the cost of NHPs and the difficulty in handling them in high-containment laboratories, especially for the screening of new vaccine candidates and antivirals. Also, the NHP model is more susceptible to developing a more severe disease than humans. Studies are further limited by the low numbers of human clinical isolates used in animal models. Immunocompromised mice, such as interferon receptor or STAT1 knockout mice, develop lethal infections but are not ideal for testing vaccines or characterizing immune responses (6, 8). Guinea pigs are the most commonly used rodent model for LF study. The inbred guinea pig strain 13 was historically used as a lethal challenge model of LF, though they are not currently commercially available (9, 10). Outbred strain Hartley guinea pigs mostly do not exhibit consistent morbidity and lethality without viral adaptation of laboratory strains through serial passage in guinea pigs (11). Animal models that are easy to use for research and that can utilize currently circulating human Lassa virus strains are important for the accelerated development of vaccines or therapeutics.

Here we show that the LASV isolate LF2384-NS-DIA-1 (LF2384), which was directly isolated from a serum sample of a fatal human LF case in the 2012 Sierra Leone outbreak and which has been reported to cause lethal infection in STAT1 knockout mice (8), causes uniformly lethal infection in Hartley guinea pigs and may be useful for testing vaccines and antivirals in future studies.

## 

### Pathogenicity of LASV LF2384 in guinea pigs.

**FIG 1:**
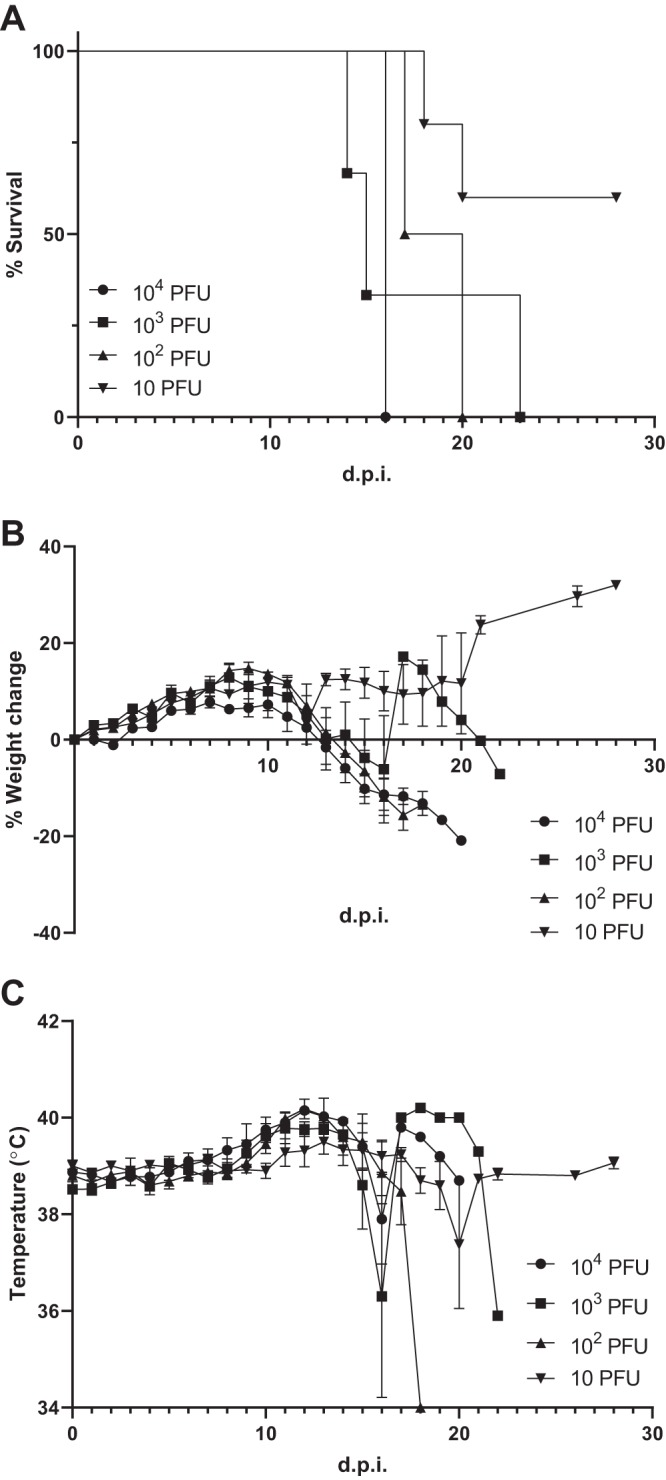
Survival, body weight, and temperature changes of LASV LF2384-inoculated guinea pigs. Five guinea pigs were inoculated with 10^4^, 10^3^, 10^2^, or 10 PFU of LASV LF2384 intraperitoneally and monitored for survival, body weight, and body temperature for 28 days postinoculation (d.p.i.). (A) Survival curve, (B) percentage weight change, and (C) body temperature. In panels B and C, values are means ± standard errors (error bars).

All guinea pigs inoculated with 10^4^, 10^3^, or 10^2^ PFU of LASV LF2384 (five mice in each group) and two of five guinea pigs inoculated with 10 PFU of LASV LF2384 succumbed to disease at 13 to 22 days postinoculation (dpi). The mean survival times with standard errors for the groups inoculated with 10^4^, 10^3^, and 10^2^ PFU are 17.6 ± 1.0, 16.4 ± 1.5, and 17.0 ± 0.3 dpi, respectively (Fig. 1A). The calculated 50% lethal dose (LD_50_) is <14.7 PFU. Animals inoculated with the virus started losing weight and developed a fever, a body temperature greater than 40°C, at 10 dpi (Fig. 1B and C). Animals began showing scruffy coats at 12 dpi and developed lethargy and loss of appetite at 13 dpi. None of the inoculated guinea pigs showed neurological symptoms prior to euthanasia. Prior to succumbing to disease, guinea pigs developed hypothermia. The three survivors inoculated with 10 PFU of LASV LF2384 showed slight weight loss at 10 to 12 dpi but recovered afterwards. None of the survivors presented with fever throughout the experiment. At the time of necropsy, small spleens were observed by gross pathology.

### Viral load and hematology of guinea pigs inoculated with LASV LF2384.

**FIG 2:**
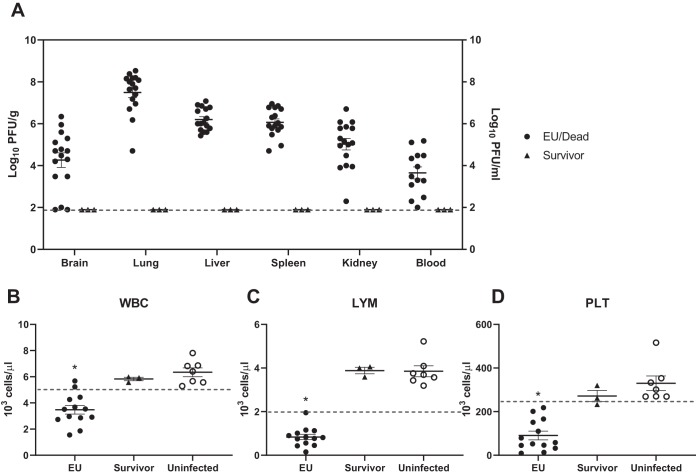
Virus titers and hematology of guinea pigs inoculated with LASV LF2384. When guinea pigs were euthanized (EU) or dead, organ and blood samples were collected, if available. (A) Homogenates of organs or whole-blood samples were used to titrate virus by plaque assay. The broken line indicates the detection limit (<2.0 log_10_ PFU/g for organs, <2.0 log_10_ PFU/ml for blood). Each symbol represents the value for an individual guinea pig. The means ± standard errors (error bars) for the guinea pig organs are shown. (B to D) Blood samples were collected from guinea pigs inoculated with LASV LF2384, and hematological parameters were measured. The numbers of white blood cells (WBC) (B), lymphocytes (LYM) (C), and platelets (PLT) (D) are shown. The broken lines indicate the lower limit of the normal range. Each symbol represents the value for an individual guinea pig. The means ± standard errors for the groups are shown. Statistical analyses were performed using Kruskal-Wallis test following Dunn’s multiple-comparison test (*, *P* < 0.01).

At the end of the study, brain, lung, liver, spleen, kidney, and blood samples were collected and used for determining viral load. Organ samples from 16 dead/euthanized guinea pigs and 3 survivors, as well as blood samples from 13 dead/euthanized guinea pigs and 3 survivors, were available to use for virus titration. All lung, liver, spleen, kidney, and blood samples and 14/16 brain samples collected from guinea pigs that succumbed to the disease had detectable virus titers (Fig. 2A). No virus was detected in survivors. Geometric mean virus titers detected from animals euthanized due to disease in the brain, lung, liver, spleen, and kidney were 4.60, 7.49, 6.20, 6.06, and 5.02 log_10_ PFU/g, respectively. The geometric mean titer from blood samples was 3.64 log_10_ PFU/ml. There was no correlation between time of death and viral load.

At the time of euthanasia, collected blood samples were used for hematology. Blood samples from uninfected guinea pigs were used as a negative control. The numbers of white blood cells (WBC), lymphocytes (LYM), monocytes, neutrophils, red blood cells, platelets (PLT), hemoglobin level, and hematocrit were determined. WBC, LYM, and PLT from guinea pigs that succumbed to disease were abnormally low and significantly lower than those of surviving guinea pigs (*P* < 0.01) (Fig. 2B to D). The other hematological parameters were within normal ranges, and no significant differences were observed (see Table S1 in the supplemental material).

10.1128/mSphere.00428-19.1 TABLE S1 Hematology data. At the time of euthanasia (*n* = 13) or at the end of the study (*n* = 3), blood samples were collected for hematology. Uninfected guinea pig blood was used for negative control (*n* = 8). Whole-blood samples collected in EDTA tubes were used for standard hematologic analysis using a VETSCAN HM5 (ABAXIS) according to the manufacturer’s instructions. Means and standard deviations are shown. Download Table S1, DOCX file, 0.02 MB. Copyright © 2019 Maruyama et al. 2019 Maruyama et al. This content is distributed under the terms of the Creative Commons Attribution 4.0 International license.

Hartley guinea pigs usually do not develop consistent morbidity upon LASV infection; even after high-dose infection with the laboratory strain Josiah, only approximately 30% of infected animals develop severe disease (10). It is noteworthy that a recent guinea pig-adapted LASV Josiah strain has been used in Hartley guinea pigs for antiviral studies with 100% lethality (11). LASV LF2384 belongs to lineage IV of LASV, which is endemic in West African countries with the exception of Nigeria, similar to the Josiah strain. The LASV Josiah strain is commonly used for LF studies; however, it is difficult to track the passage history of this strain as it was isolated in the 1970s (12). Our novel guinea pig model of LF using LASV LF2384 demonstrates 100% lethality with 100 PFU inoculation without any need for virus adaptation. It has been reported that LASV LF2384 causes lethal infection in Stat1 knockout mice (8). In this study, it was revealed that this virus was pathogenic not only in immunocompromised animals but also in immunocompetent guinea pigs. The LD_50_ of LASV LF2384 in guinea pigs is less than 15 PFU. The mean survival times of groups inoculated with 10^4^, 10^3^, and 10^2^ PFU are around 17 dpi, suggesting that this model has good reproducibility as a lethal model. In this model, infected guinea pigs present with leukopenia, lymphopenia, and thrombocytopenia with high viral loads in organs, which is similar to human LF case reports. These features provide our model several advantages for its application in antiviral and pathogenicity studies. Additionally, LASV LF2384 was isolated directly from a human clinical sample. This is the first report of a clinically isolated strain of LASV causing lethal infection in outbred guinea pigs.

Although further studies are necessary to reveal the molecular mechanisms underlying its pathogenicity in guinea pigs, this new model of LF may play an important role in acceleration of LF research and the testing of vaccine and therapeutic candidates.

### Cells and virus.

Vero cells were maintained within Dulbecco’s modified Eagle’s medium supplemented with 10% fetal bovine serum (FBS), 1% penicillin-streptomycin, and l-glutamine. LASV strain LF2384, which belongs to lineage IV, was isolated from a fatal LF case during a 2012 outbreak in Sierra Leone (8). The virus was isolated from the serum sample in Vero cells, and virus-containing cell culture supernatant was stored in –80°C freezer until use without any additional passaging. All work with infectious LASV was performed in biosafety level 4 (BSL-4) facilities in the Galveston National Laboratory (GNL) at the University of Texas Medical Branch (UTMB) in accordance with institutional guidelines.

### Virus challenge of guinea pigs.

Five- to 7-week-old Hartley guinea pigs were purchased from Charles River. All animals were housed in animal biosafety level 2 (ABSL2) and ABSL4 facilities in the GNL at UTMB. All animal studies were reviewed and approved by the Institutional Animal Care and Use Committee at UTMB and were conducted according to the National Institutes of Health guidelines. Body temperature measurements were performed using subcutaneously implanted BMDS IPTT-300 transponders and a DAS-6007 transponder reader (Bio Medic Data Systems). Body weights were measured by using a Sartorius AY1501 digital scale. Guinea pigs were intraperitoneally inoculated with diluted LASV strain LF2384 (10^4^ to 10 PFU) in 100 μl of phosphate-buffered saline (PBS) and monitored daily for 28 days after inoculation. Animals were humanely euthanized once they showed neurological symptoms such as paralysis or were unable to access their food or water and/or lost more than 20% of their body weight based on their baseline measurement at the time of inoculation. Blood and organs (brain, lung, liver, spleen, and kidney) were collected at the end of the study.

### Hematology.

Whole-blood samples collected in EDTA tubes were used for standard hematologic analysis using a VETSCAN HM5 (ABAXIS) according to the manufacturer’s instructions.

### Virus titration.

Virus titers were determined by plaque assay. Briefly, confluent Vero cells in 12-well plates were inoculated with virus diluted 10-fold and incubated for 1 h at 37°C with 5% CO_2_. After the inoculum was removed and the wells were washed, each well was overlaid with minimum essential medium containing 2% FBS, 1% penicillin-streptomycin, and 0.6% tragacanth (Sigma). Following a 5-day incubation, cells were fixed with 10% formalin and stained with crystal violet. Viral titers were represented as PFU.

### Data availability.

Sequences have been deposited in GenBank under accession no. MN275172 and MN275173.
