# Supplementary material for: Lethal Infection of Lassa Virus Isolated from a Human Clinical Sample in Outbred Guinea Pigs without Adaptation
Source: mSphere. 2019 Sep 25;4(5):e00428-19. doi: 10.1128/mSphere.00428-19 (PMC6763766; doi:10.1128/mSphere.00428-19)
Supplement: TABLE S1 [file mSphere.00428-19-st001.docx]

| Table S1. Hematology data | | |  |  |  |  |  |  |
| --- | --- | --- | --- | --- | --- | --- | --- | --- |
|  |  | **Normal  range** | **Euthanized** | | **Survivors** | | **Uninfected** | |
|  |  |  | **mean** | **S.D.** | **mean** | **S.D.** | **mean** | **S.D.** |
| **WBC** | **(10^3^/µl)** | 5.00-17.00 | 3.48 | 1.20 | 5.83 | 0.23 | 6.07 | 1.14 |
| **LYM** | **(10^3^/µl)** | 2.00-15.00 | 0.84 | 0.44 | 3.88 | 0.26 | 3.63 | 0.88 |
| **MON** | **(10^3^/µl)** |  | 0.14 | 0.13 | 0.05 | 0.00 | 0.26 | 0.18 |
| **NEU** | **(10^3^/µl)** | 1.00-11.00 | 2.50 | 1.18 | 1.90 | 0.04 | 2.18 | 0.52 |
| **LYM %** | **(%)** |  | 26.50 | 14.18 | 66.53 | 1.86 | 59.43 | 6.16 |
| **MON %** | **(%)** |  | 4.40 | 4.01 | 0.90 | 0.00 | 4.56 | 3.95 |
| **NEU %** | **(%)** |  | 69.14 | 14.46 | 32.57 | 1.86 | 36.01 | 5.57 |
| **RBC** | **(10^6^/µl)** | 3.50-7.00 | 5.76 | 0.36 | 6.25 | 0.40 | 6.74 | 0.95 |
| **HGB** | **(g/dl)** | 11.00-18.00 | 13.62 | 0.95 | 14.60 | 0.61 | 14.35 | 1.49 |
| **HCT** | **(%)** | 35.00-55.00 | 44.79 | 2.67 | 49.03 | 1.17 | 53.72 | 7.47 |
| **MCV** | **(fl)** | 70.00-95.00 | 77.77 | 1.30 | 81.33 | 3.21 | 80.88 | 3.27 |
| **MCH** | **(pg)** | 23.00-27.00 | 23.64 | 0.79 | 24.17 | 1.33 | 21.61 | 3.43 |
| **MCHC** | **(g/dl)** | 28.00-38.00 | 30.40 | 0.88 | 29.77 | 0.55 | 26.79 | 4.52 |
| **RDWc** | **(%)** |  | 15.95 | 1.07 | 14.57 | 0.90 | 17.54 | 1.38 |
| **RDWs** | **(fl)** |  | 47.18 | 3.32 | 44.80 | 5.33 | 53.43 | 5.13 |
| **PLT** | **(10^3^/µl)** | 250-850 | 90.77 | 71.30 | 271.67 | 44.30 | 313.13 | 95.05 |
| **MPV** | **(fl)** |  | 8.42 | 0.53 | 6.60 | 0.26 | 6.84 | 0.27 |
| **PCT** | **(%)** |  | 0.08 | 0.06 | 0.18 | 0.04 | 0.22 | 0.07 |
| **PDWc** | **(%)** |  | 34.25 | 2.40 | 30.13 | 1.55 | 30.15 | 1.12 |
| **PDWs** | **(fl)** |  | 11.72 | 2.22 | 7.90 | 0.87 | 7.91 | 0.67 |

WBC: total white blood cell count. LYN: lymphocyte count. MON: monocyte count. NEU: neutrophil count. LYM%: lymphocyte percentage. MON%: monocyte percentage. RBC: red blood cell count. HGB: hemoglobin. HCT: hematocrit. MCV: mean corpuscular volume. MCH: mean corpuscular hemoglobin. MCHC: mean corpuscular hemoglobin concentration. RDWc, RDWs: red cell distribution width, coefficient of variation. PLT: platelet count. PCT: platelet crit. MPV: mean platelet volume. PDWc, PDW: platelet distribution width, coefficient of variation.
